# Effect of maternal diet on placental development, uteroplacental blood flow, and offspring development in beef cattle

**DOI:** 10.21451/1984-3143-AR2018-0050

**Published:** 2018-08-03

**Authors:** Kimberly A. Vonnahme, Amelia R. Tanner, Manuel Alexander Vasquez Hildago

**Affiliations:** 1 North Dakota State University, Department of Animal Sciences, Fargo, ND, USA; 2 Current address: Zoetis, Parsippany, NJ 07054, USA

**Keywords:** maternal nutrition, placenta, umbilical blood flow, uterine blood flow.

## Abstract

Considerable effort has been made to understand how nutrition influences livestock health and productivity during the postnatal period. Moreover, while efforts have been made to understand how nutrition impacts many different aspects of female reproduction, there is a growing body of literature that focuses on how maternal nutrition impacts the developing fetus. Providing adequate nutrition during pregnancy is important for maternal health and well- being, as well as conceptus development. Proper establishment of the placenta is important for fetal survival. However, placental adaptations to inadequate maternal nutrition, or other stressors, are imperative for fetal growth to be optimal. By understanding how the maternal environment impacts uterine and umbilical blood flows and other uteroplacental hemodynamic parameters, we can better implement supplementation strategies to protect the developing offspring. This review focuses on how maternal nutrition affects conceptus growth in sheep and beef cattle and offspring performance after birth.

## Introduction

The maternal system can be influenced by many different extrinsic factors, including nutritional status, which can program nutrient partitioning and ultimately growth, development and function of the major fetal organ systems (Wallace, 1948; Godfrey and Barker, 2000; Wu *et al*., 2006). The trajectory of prenatal growth is sensitive to direct and indirect effects of maternal environment, particularly during early stages of embryonic life (Robinson *et al*., 1995), the time when placental growth is exponential. Understanding the impacts of the maternal environment on placental growth and development is especially relevant as the majority of mammalian livestock spend 35-40% of their life within the uterus, being nourished solely by the placenta. Moreover, pre-term delivery and fetal growth restriction are associated with greater risk of neonatal mortality and morbidity in livestock and humans. Offspring born at an above average weight have an increased chance of survival compared with those born at a below average weight in all domestic livestock species, including the cow and ewe. Just as growth restricted human infants are at risk of immediate postnatal complications and diseases later in life (Barker *et al*., 1993; Godfrey and Barker, 2000), there is increasing evidence that production characteristics in our domestic livestock may also be impacted by maternal diet (Wu *et al*., 2006). Some of the complications reported in livestock include increased neonatal morbidities and mortalities (Hammer *et al*., 2011), intestinal and respiratory dysfunctions, slow postnatal growth, increased fat deposition, differing muscle fiber diameters and reduced meat quality (reviewed in Wu *et al*., 2006).

The objective of this review is to highlight maternal adaptation to pregnancy, some of our laboratories investigations on how maternal nutrition can impact uterine and/or umbilical blood flow in cattle and sheep, as well as to highlight beef cattle studies that have investigated different supplementation strategies that may influence carcass development or fertility of the offspring.

## Cardiovascular responses of the pregnant dam

Maternal cardiovascular capacity changes dramatically during pregnancy, with decreases in systemic arterial blood pressure and vascular resistance and increases in cardiac output, heart rate, heart stroke volume, and blood volume (Magness, 1998). Mean arterial pressure decreases in early pregnancy and persists throughout gestation in several mammalian species. The decrease in arterial pressure (~5 to 10% decrease) is minor compared to the approximate 20 to 30% decrease in total peripheral vascular resistance. Maternal cardiac output has been shown to increase as much as 30 to 40% in pregnant *vs.* non-pregnant ruminants (Magness, 1998).

An adequate blood volume increase is necessary in order to protect the mother and fetus from the deleterious effects of a reduced venous blood return and cardiac output (Pritchard, 1965; Torgersen and Curran, 2006). While it is well established why maternal blood volume needs to increase, understanding how blood volume increases is still under study. There are currently two theories that attempt to explain blood volume expansion: the decreased vascular resistance theory and the endocrine theory.

The decreased vascular resistance theory (Schrier and Briner, 1991; Duvekot *et al*., 1993) states that when the female becomes pregnant, a new vascular system is added to her pre-existing vascular system, which decreases the total vascular resistance of the mother’s cardiovascular system. A decrease in total vascular resistance in turn increases maternal heart rate, activating the plasma volume regulating mechanisms in the liver, kidneys, and adrenal glands (Schrier and Briner, 1991; Duvekot *et al*., 1993). As plasma volume increases, blood volume increases as well.

The endocrine control theory (Longo, 1983) suggests a fetal-placental influence on blood volume in the pregnant female. As gestation advances, the fetal adrenal glands increase in size and their production of dehydroepiandrosterone, stimulates estradiol production in the mother (Longo, 1983). Estradiol stimulates the renin-angiotensin system, ultimately increasing plasma volume (Longo, 1983). Moreover, as placental size increases, there is increased somatomammotropin (i.e. placental lactogen) and progesterone production (Longo, 1983). These two hormones stimulate the production of erythropoietin in the mother, ultimately stimulating erythrocyte production (Longo, 1983).

In women, failure to adequately increase blood volume during pregnancy has been linked to pregnancy- induced toxemia (preeclampsia), fetal growth restriction, and premature labor (Goodlin *et al*., 1981). An inadequate function of the mechanisms necessary to increase blood volume in a state of decreased vascular resistance could consequently increase heart rate and produce vasoconstriction (Lund and Donovan, 1967; Goodlin *et al*., 1981). To our knowledge, there is little information available on how maternal blood volume is affected in our livestock species, let alone how nutritional or other environmental factors may be altering plasma volume expansion. In sheep, maternal nutrient restriction resulted in a decreased plasma volume, and reduced concentration of angiotensinogen, compared to well-fed ewes (Dandrea *et al*., 2002). Upon realimentation, previously restricted ewes had greater plasma volume compared to the ewes that were well-fed continuously, and authors concluded that these adaptations may have assisted in placental growth and function (Dandrea *et al*., 2002). More work is needed to determine if placental insufficiencies observed in various nutritional models are associated with the inability for the maternal blood volume to expand adequately in times of suboptimal nutritional resources.

## The ruminant placenta

Unlike most eutherians, ruminant livestock have non-invasive placentas. Gross morphology of the ruminant placenta is termed cotyledonary, where the fetal placenta attaches to discrete site on the uterine wall called caruncles (Ford, 1999). The placental membranes attach at these sites via chorionic villi in areas termed cotyledons. The caruncular-cotyledonary unit is called a placentome and is the primary functional area of physiological exchanges between mother and fetus. Microscopically, livestock species have epitheliochorial placentation, with six cellular layers separating maternal and fetal blood. Some argue that ruminant placentas are better classified as syndesmochorial due to their formation of giant trophoblast cells by chorionic and uterine epithelia (Wooding and Wathes, 1980; Hoffman *et al*., 1986). The binucleate cells migrate during implantation and fusion with the caruncular epithelium to cause a shift from cellular to maternofetal syncytial plaques (Wooding, 1984; Wango *et al*., 1990). Binuclear cells have two principal functions: 1) to create the maternofetal syncytium necessary for proper implantation and 2) to promote placentomal growth, as well as to produce and deliver a variety of steroid and protein hormones (Wooding, 1992).

In the ewe, the growth of the cotyledonary mass is exponential during the first 70 to 80 days of pregnancy, thereafter slowing markedly until term (Stegeman, 1974). In contrast, the placental growth in the cow progressively increases throughout gestation (Vonnahme *et al*. 2007; Funston *et al*., 2010). Perhaps, these alterations in growth patterns in the sheep and cow placenta help explain the change of capillary area density (*i.e.*, a blood flow related measure; Borowicz *et al*., 2007) that exist from mid- to late-gestation (Reynolds *et al*., 2005). While sheep placentas remain relatively similar in weight from mid- to late-gestation, their caruncular and cotyledonary capillary area density increase ~200 and 400%, respectively (Reynolds *et al*., 2005; Borowicz *et al*., 2007). Bovine placentas exhibit relatively modest changes in capillary area density (compared to sheep) from mid- to late-gestation with capillary area density in caruncular tissue decreasing ~30% and cotyledonary tissue increasing ~190%, with caruncular and cotyledonary tissue weights increasing ~530 and ~650%, respectively (Vonnahme and Lemley, 2011).

## Prenatal dietary impacts on conceptus development

This idea that improper maternal nutrition in late pregnancy decreases offspring performance had already been suggested by Wallace (1948) and because the majority (75%) of ovine fetal growth occurs over the last two months of gestation (Robinson *et al*., 1977) it is logical to understand why adequate maternal nutrition during late gestation is critical for maintaining fetal growth. These deleterious effects observed in offspring of nutrient restricted dams are not limited to neonatal growth and health, rather this programmed effect has a profound effect on lifelong growth and increases the likelihood of developing non-communicable diseases later in life (Barker *et al*., 1993; Godfrey and Barker, 2000). In addition to the evident need for developmental programming research in biomedical science, improper maternal nutrition during pregnancy also has profound consequences on livestock offspring health and performance (Wu *et al*., 2006). Compounding on offspring altered growth trajectories, improper maternal nutrition also leads to a decrease in fertility as well as the carcass quality of the offspring, including altered fat deposition, muscle fiber type and reduced meat quality (Wu *et al*., 2006). To understand how maternal diet can influence offspring fertility, fetal ovaries collected from late pregnant ewes that experienced a 40% nutrient restriction from mid to late gestation, had primordial follicles with a decreased cellular proliferation rate compared to ovaries from fetuses of adequately fed ewes (Grazul-Bilska *et al*., 2009). This decreased proliferation in the primordial follicle may impact future follicular activity, fertility, and reproductive longevity of the female offspring. Unfortunately, data do not indicate the reproductive success of these offspring. It has been previously proposed maternal protein supplementation may affect oocyte quality or early embryonic formation, resulting in fewer calves born during the first 21 days of the calving season (Martin *et al*., 2007). Furthermore, heifers born from dams protein supplemented during the last third of pregnancy had an increased pregnancy rate compared to heifers from non-supplemented dams (Martin *et al*., 2007). Fewer heifers from non-supplemented dams attained puberty before the first breeding season compared with heifers from supplemented cows in a subsequent study (Funston *et al*., 2010). Additionally, in rats where dams were protein restricted during pregnancy, female pups had a delay to vaginal opening (i.e. puberty) and time to first estrus compared to control dams (Guzman *et al*., 2006).

Several authors have established that many of the models of placental insufficiency are, in part, due to reduced placental vascularity and uterine or umbilical blood flows (reviewed in Owens *et al*., 1986; Fowden *et al*., 2006; Vonnahme and Lemley, 2011; Burton and Fowden, 2012). When placental growth is restricted in ewes, umbilical and uterine blood flows are reduced, limiting fetal growth (Owens *et al*., 1986). Recently, in our laboratories, we have investigated if placental vascularity and uterine/umbilical blood flows are impacted by different maternal dietary treatments. In sheep, while we have observed reductions in umbilical blood flow (Lemley *et al*., 2012) and increases in arterial indices of resistance (Lekatz *et al*., 2013), we have not observed alterations in placental capillary densities (Lekatz *et al*., 2010; Eifert *et al*., 2015). Umbilical blood flow of singleton fetuses was reduced after 30 days of receiving a 40% global nutrient restriction compared to adequately fed control ewes (Lemley *et al*., 2012). This reduction in umbilical blood flow remained through late gestation (day 130). In beef cattle, we initially hypothesized that, similar to sheep, reductions in intake would lead to reductions in uterine blood flow. In contrast, we observed that during a 110 day nutrient restriction (i.e., 40% of the control diet), uterine (Camacho *et al*., 2014) and umbilical (Camacho *et al*., 2018) blood flows were similar. Interestingly, upon realimentation, blood flow to the ipsilateral horn increases (Camacho *et al*., 2014). While our work has been done with global nutrient restriction and realimentation, Perry *et al*. (1999) reported that protein restriction during the first trimester of pregnancy followed by increased protein concentration during the second trimester enhances placental development and fetal growth. Increased dry matter intake has been linked to enhanced maternal insulin-like growth factor-1 during late pregnancy (Lemley *et al*., 2014). If exogenous insulin-like growth factor-1 is administered to the dam, there is increased glucose and amino acid uptake by both fetal and maternal tissues (Sferruzzi-Perri *et al*., 2007). Uteroplacental blood flow is undoubtedly associated with fetal growth and development; however, specific nutrient transport across the feto-placental unit rely not only on adequate blood flow but also on adequate nutrient transporter densities.

## The timing and duration of inadequate maternal nutrition

If there is a nutritional inadequacy during pregnancy, the timing, duration, and severity of that restriction greatly impacts fetal development. While most fetal growth occurs during late gestation (Robinson, 1977), inadequate nutrition during early gestation can have profound effects on placental development, vascularization, and fetal organogenesis (Funston *et al*., 2010). Adult sheep that are nutrient restricted during late gestation experience a 17 to 32% decrease in uterine blood flow, decreased caruncular capillary area density as well as reduced fetal weight (Anthony *et al*., 2003; reviewed in Reynolds *et al*., 2006). Additionally, underfed multiparous cows that were nutrient restricted during mid and late gestation experienced significant reductions in calf birth weights (reviewed in Greenwood and Café, 2007).

Underfed adolescent animals often respond differently than mature animals. Heifers that were nutrient restricted during gestation experienced more extreme birth weight reductions in their calves than mature beef cows (reviewed in Greenwood and Café, 2007). Additionally, placental alterations can occur due to altered maternal nutrition during early-to mid- gestation without impacting fetal weights (Rasby *et al*., 1990). The bovine placenta also appears to be sensitive to protein supplementation during early- to mid-gestation but calf birth weight was not impacted (Perry *et al*., 1999; Perry *et al*., 2002). However, because the bovine placenta continues to grow throughout gestation (Prior and Laster, 1979; Ferrell, 1989) it has been suggested, that the bovine placenta is not as sensitive to nutritional deficiencies as the ovine placenta (Ferrell, 1989; Greenwood and Café, 2007). While there are many brilliant reviews written about the impacts of maternal nutrition on ovine conceptus development, the focus of this review will be how maternal nutrient restriction and supplementation strategies impact calf development.

## Nutrient restriction in the beef cow

Global nutrient restriction on the dam during various stages of gestation can impair placental function and calf growth depending on the severity of the restriction, timing of nutritional insult, parity, and pregnancy type (Tables 1 and 2). In comparison to sheep, the developing bovine fetus is more susceptible to alterations in myogenesis and adipogenesis when subjected to maternal nutrient restriction during mid- to late-gestation (Greenwood and Café, 2007) whereas nutritional restrictions during early pregnancy can have subtle effects on organogenesis, causing long-term health complications (Greenwood and Bell, 2003; Bell *et al*., 2005). Pre-breeding management of heifers influenced uterine hemodynamics as low input (fed to 45 to 55% of mature body weight at breeding) females had increased uterine blood flow (adjusted for maternal body weight) compared to conventionally fed heifers, with authors hypothesizing the increased uterine blood flow compensated for inadequate heifer body reserves (Cain *et al*., 2017; Table 2).

When heifers are not provided adequate metabolizable energy or crude protein during early- to mid-gestation, fetal weights are reduced (Micke *et al*., 2010). Interestingly, fetuses from restricted heifers had increased umbilical diameters compared to fetuses from adequately-fed heifers (Sullivan *et al*., 2009; Micke *et al*., 2010; Table 3). If an increased umbilical diameter equates to the potential for increased umbilical blood flow, this may serve as a compensatory mechanism to support fetal growth, as evidenced by greater fetal abdominal circumference and crown-nose length and thoracic diameter (Sullivan *et al*., 2009; Micke *et al*., 2010). Our laboratory has also demonstrated that nutrient restriction during early pregnancy (day 30 to 85) in multiparous cows results in calves being larger, which is probably due to the larger placentome mass observed in those restricted cows (Camacho *et al*., 2018; Table 2). Others have reported that heifers nutrient restricted to 60% of their NRC requirements in early gestation (conception to day 60 gestation) had normal calf birth weights and placental weights (Spiegler *et al*., 2014).

When global nutrient restriction occurs during mid- to late-pregnancy (day 118 to term), dams progressively lost body condition and had reduced calf birth weights (Freetly *et al*., 2000). Similarly, Corah *et al*. (1975) reported that heifers nutrient restricted to 57% of their NRC requirements during late gestation had a reduction in fetal weights. In contrast, no birth weight differences were detected when mature cows were nutrient restricted to 55% of their NRC requirements during a similar time period (Hough *et al*., 1990). Perhaps, multiparous cows have a compensatory mechanism for maintaining proper fetal growth that first time dams do not possess.

While skeletal muscle development *in utero* sets the foundation for postnatal growth performance, nutrient delivery within the fetus is shuttled to more important organs, such as the brain and heart (Bauman *et al*., 1982; Close and Pettigrew, 1990). The fetal period is crucial for skeletal muscle development, because no net increase in the number of muscle fibers occurs after birth (Glore and Layman, 1983; Greenwood *et al*., 2000; Nissen *et al*., 2003). Therefore it should not be surprising that skeletal muscle development is altered by maternal diet (Table 4). Calves from nutrient restricted beef cows have decreased average daily gain in the feed yard, fatter carcasses at 30 months of age (Café *et al*., 2006; 2009), and had reduced carcass weights (Greenwood *et al*., 2004). Gonzales *et al*. (2013) could rescue muscle fiber size and muscle progenitor cell numbers through realimentation of late- gestating beef cows that were previously nutrient restricted during early pregnancy. Heifers that only received 55% of their NRC requirements during early gestation (day 32 to 115) had calves with increased muscle fiber diameter, faster glucose clearance, but similar feed efficiency as calves from continuously well- fed dams (Long *et al*., 2010a; Long et al., 2010b). Interestingly, weaning weights, carcass composition, and carcass quality of those cattle were unaffected (Long *et al*., 2010b) possibly due to the realimentation.

By increasing the forage quality of late gestating beef cows, Underwood *et al*. (2010) improved average daily gain, increased 12th rib subcutaneous fat, and hot carcass weights of calves, although earlier differences in neonatal performance were not detected. Similarly, Mohrhauser *et al*. (2013) reported that cows fed diets exceeding their energy requirements during mid-gestation, had calves that were fatter and had lower yielding carcasses compared to offspring from dams fed a negative energy diet.

## Maternal supplementation strategies in the beef cow

Because there are many times during pregnancy when the dam only has access to low quality forages, supplementation of dietary protein and energy supplements have been examined (Tables 3, 4, and 5). Cows supplemented with dried distiller’s grains plus solubles (DDGS) during late gestation (Radunz *et al*., 2010; Gunn *et al*., 2014, 2017) as well as into early lactation (Winterholler *et al*., 2012) had increased calf birth weights, however, this effect disappeared by weaning. Conversely, Kennedy *et al*. (2016) observed increased birth weights coupled with heavier weaning weights of calves whose dams received DDGS supplementation at 0.3% body weight during late gestation (day 200 to 270). This difference is likely due to the improved roughage intakes of DDGS supplemented beef cows and their increased uterine blood flow (Kennedy *et al*., 2016). Perhaps *ad libitum* access to forage is a critical component to the response of DDGS supplementation. When cows that were fed low quality hay at 2% of body weight and supplemented with DDGS at 1.7 g/ kg of body weight, uterine blood flow was reduced (Mordhorst *et al*., 2017). Despite the decreased uterine blood flow, calf birth weights were similar (Mordhorst *et al*., 2017).

To examine effective timing of dam protein supplementation and improved forage strategies, multiparous beef cows were provided with either a protein supplement at 0.45 kg/day or no supplement during late gestation and grazed on either subirrigated meadow or cool-season grass hay during lactation (Stalker *et al*., 2006; Martin *et al*., 2007). While no differences in calf birth weight or maternal milk production were detected, calves from protein supplemented dams who grazed subirrigated meadow during lactation had heavier calves at weaning (Stalker *et al*., 2006; Martin *et al*., 2007). While no differences in carcass composition or carcass quality were detected amongst the steers (Stalker *et al*., 2006), the heifers of protein supplemented dams had improved pregnancy rates compared to heifers whose dams did not receive the supplement (Martin *et al*., 2007). In a subsequent study, steers from protein supplemented dams who grazed winter range had heavier weaning weights and improved hot carcass weight and USDA quality grades (Larson *et al*., 2009). Supplementation during late pregnancy also benefits postnatal performance if early weaning strategies are implemented (Shoup *et al*., 2015a, b), where weaning weights, average daily gain, and marbling are increased. In spite of these interesting findings, there are other investigators that report no impacts of protein supplementation, which may be due to the stage of gestation, severity of basal nutritional insult, and components of the supplement (Greenwood and Café, 2007).

**Table 1:** The effects of bovine maternal nutrient restriction on calf and placental parameters.

Parity and stage^1^	Diet^2^	Offspring sex	Change in dam body weight	Fetal/ birth weights	Placental weights^3^	Placental vascularity^4^	Reference
N; day 183- term	CON (100% NRC) or RES (55% NRC)	♂ + ♀	RES ↓	RES ↓	---	---	Corah *et al*., 1975
M; day 193- term	CON (100% NRC) or RES (57% NRC)	♂ + ♀	RES ↓	NSE	---	---	Hough *et al*., 1990
M; Mid-gestation - rebreeding	Maintain BCS (HHH); ↓ BCS 2nd trimester then ↑ (LHH); or ↓ BCS until 28 day of lactation (LLH)	♂ + ♀	HHH > LLH at calving	LLH ↓	---	---	Freetly *et al*., 2000
M; day 30-125	100 *vs*. 50% NRC from day 30- 125 then 100% NRC to day 250	♂	---	NSE	NR COT and CAR ↓ @ day 125 and 250	NR ↑ Akt & ERK1/2 phosphorylation @ day 125	Zhu *et al*., 2006
M; day 30-125	100 *vs*. 50% NRC from day 30- 125 then 100% NRC to day 250	♀	RES ↓ then realimented BCS 5.75	NSE	---	Realimentation ↑ CAR CSD; ↓ COT CAD and CSD	Vonnahme *et al*., 2007
M; day 30-125	100 *vs*. 50% NRC from day 30- 125 then 100% NRC to day 250	♀	RES ↓ then realimented BCS 5.75	NR- IUGR ↓ day 125	NR-IUGR ↓ COT wt	---	Long *et al*., 2009

1 Parity: M, multiparous; N, nulliparous; Stage: stage of pregnancy.

2 Description of diets; NRC, National Research Council; BCS, body condition score.

3 CAR, caruncle; COT, cotyledon; IUGR, intrauterine growth restricted.

4 CSD, capillary surface density; CAD, capillary area density.

**Table 2:** The effects of bovine maternal nutrient restriction on uterine hemodynamics and birth weights.

Parity and stage^1^	Diet^2^	Offspring sex	Change in dam body weight	Uterine blood flow	Placental weights	Fetal/ birth weights	Reference
M; early through late (day 30 to 85, early; day 85 to 140, mid; day 140 to 254, late)	Early: C (100% NRC), R (60% NRC); Mid: CC, RR, RC; Late: CCC, RRR, RCC	♂ + ♀	day 85 = R ↑BW; day 140 = RR ↓BW; day 254 = RCC ↑BW	RCC > RRC=CCC	R and RR ↑ placentome number and weight compared to C, CC	d 85 = R ↑ *vs*. C	Camacho *et al*., 2018
M; early through mid	CON (100% NRC); RES (60% NRC) until day 140 then realimented	♂ + ♀	RES ↓	NSE during restriction; RES after realimentation ↑ ipsi UBF *vs*. CON	---	---	Camacho *et al*., 2014
P; Pre-breeding management until day 45	Spring (S) or Fall (F) fed LOW input or CONventional	♂ + ♀	CONǀS > CONǀF > LOWǀF > LOWǀS	Spring ↑UBF; LOW ↑ adjusted UBF	---	Spring ↑ *vs*. Fall	Cain *et al*., 2017

1 Parity: M, multiparous; P, primiparous; Stage: stage of pregnancy.

2 Description of diets; NRC, National Research Council; BCS, body condition score.

3 UBF, uterine blood flow; Ipsi, ipsilateral uterine horn. NSE, no significant effect; ---, not reported.

**Table 3:** The effects of bovine maternal nutrient supplementation on uterine hemodynamics, placental parameters, and neonatal performance.

Parity and stage^1^	Diet^2^	Offspring sex	Supplement	Offspring weights	Uterine/ umbilical blood flow^3^	Calf weaning weight	Reference
P; Early and mid-gestation; realimentated late gestation	HIGH (High ME and CP) or LOW (Low ME and CP) fed early and/or mid gestation (2 x 2 factorial)	♂ + ♀	---	HIGH-HIGH > LOW-LOW	Umbilical cord diameter ↑ high protein (first trimester); ↓ high protein (2nd trimester)	---	Micke *et al*., 2010
M; day 177 - 13 day lactation	5 supplementation diets: DDGSLow, DDGSIntermediate, DDGSHigh, positive status (POS) and negative (NEG)	♂ + ♀	DDGSL (0.77kg/day); DDGSI (1.54 kg/day); DDGSH (2.31kd/day)	DGSH > NEG	---	NSE	Winterholler *et al*., 2012
M; day 200-270	CON = 90% corn stover, 10% corn silage basal diet fed ad libitum; SUP = CON diet + DDGS at 0.3% body weight	♂ + ♀	DDGS at 0.3% body weight	SUP ↑ *vs*. CON	SUP ↑UBF and HR, ↓PI *vs*. CON	SUP ↑ *vs*. CON	Kennedy *et al*., 2016
M; day 170- 234	Hay at 2% BW (CON), DDGS+ hay (SUP)	♂ + ♀	DDGS at 1.7 g/kg BW	NSE	SUP ↓ UBF *vs*. CON	NSE	Mordhorst *et al*., 2017

1 Parity: M, multiparous; P, primiparous; Stage: stage of pregnancy.

2 Description of diets; ME, metabolizable energy; CP, crude protein; DDGS, dried distillers grains plus solubles;

3 UBF, uterine blood flow. NSE, no significant effect; ---, not reported.

**Table 4:** The effects of bovine maternal nutrient restriction during gestation on offspring postnatal performance and carcass parameters.

Parity & Stage^1^	Diet	Offspring sex	Fetal/ Birth weights	Weaning weights	Feed efficiency	HCW	USDA QG	USDA YG	12th rib fat	Study
M; day 80- term	High (H) or Low (L) plane of nutrition during gestation. Cross-over at birth	♂ + ♀	H ↑	NSE	H ↑ ADG and DMI	---	NSE	NSE	NSE	Café *et al*., 2006, 2009
M; day 120 - 180	Native pasture (protein restricted) vs. improved pasture (IP)	♂	NSE	IP ↑ vs. native pasture	IP ↑ ADG vs. native pasture	IP ↑ vs. native pasture	NSE	NSE	IP ↑ *vs*. native pasture	Underwood *et al*., 2010
M; day 84 -175	Dormant range = positive energy status (PES) vs. 80% of NRC = negative energy status (NES)	♂ + ♀	---	---	---	NSE	NSE	PES ↓ vs. NES	PES ↑ *vs*. NES	Mohrhauser *et al*., 2013

1 Parity: M, multiparous; Stage: stage of pregnancy. NSE, no significant effect; ---, not reported.

**Table 5:** The effects of bovine maternal supplementation during gestation on offspring postnatal performance and carcass parameters.

Parity & Stage^1^	Basal diet	Supplement	Experimental design	Weaning weight	Feed efficiency	Hot carcass weight	USDA quality grade	USDA yield grade	12th rib fat	Reference
M; Late gestation - early lactation	Meadow or Hay forage	0.45 kg/day with 42% CP (PS) or no supplement (NS)	2 x 2 factorial; forage x supplement	M-PS ↑	NSE	NSE	NSE	NSE	NSE	Stalker *et al*., 2006
M; Nov- Mar	Winter range (WR) vs. corn residue (CR)	Protein supplement (PS) or none (NS)	2 x 2 factorial; forage x supplement	WR-PS > WR-NS	NSE	WR-PS ↑	PS ↑ vs. NS	NSE	NSE	Larson *et al*., 2009
M; day 160 - 275	Hay; or 4.1 kg DDGS; 5.3 kg corn	1 kg pelleted supplement for DDGS and Corn		Corn > hay	NSE	NSE	Corn ↓	NSE	NSE	Radunz *et al*., 2010, 2012
M; day 45- 185	CON (100% NRC), NR (70% NRC); NRP (70% NRC + essential amino acids)	NRP treatment only = essential amino acids supplement to equal CON		NSE	---	NSE	NR ↑ adipocyte	NR ↓	NSE	Long *et al*., 2012
M; day 180- term; early	Pasture fed; normal or early wean	Low supplement = 2.16 kg/day, high supplement = 8.61 kg/day or no supplement (NS)	2 x 3 factorial; time of weaning x supplement	Low supplement > no supplement when early weaned	Low supplement in normal wean ↑	NSE	High supplement > No supplement	NSE	NSE	Shoup *et al*., 2015a, b
P; day 142- term	CON = hay; HI = hay + DDGS: LO = hay + corn- gluten	HI- DDGS (0.83 kg/day); LO- corn gluten (0.83 kg/day)		NSE	CON ↑DMI and RFI	NSE	CON ↑	CON < LO	CON > LO	Summers *et al*., 2015 a, b

1 Parity: M, multiparous; P, primiparous; Stage: stage of pregnancy. NSE, no significant effect; ---, not reported.

## Conclusions

It is important that we continue to understand the capabilities of the maternal system to various stressors that occur during pregnancy. With increased knowledge of how the dam responds to stressful nutritional paradigms (i.e., inappropriate nutrient supply, conditional increased nutrient demand, specific nutrient imbalances, etc.), we may have the chance to increase the welfare for the dam as well as the offspring throughout their productive life. It appears that the multiparous beef cow is quite resilient to many different nutritional stressors compared to the first calf heifer. While this may be due to age, previous pregnancies, and the increased hormonal profiles associated with being pregnant, surely alters the uterine vasculature and its ability to nourish subsequent calves. Moreover, it appears that the placenta is responsive to inadequate nutrition, so that in times when nutrients are suboptimal, it simply grows to increase its surface area of attachment. Knowing the placental adaptations that the multiparous beef cow is capable of may explain why in many models of nutrient restriction, calves do not have negative impacts on their carcass phenotypes.

Protein supplementation, when forage is not limiting, appears to enhance uterine blood flow, which in turn may allow greater nutrient delivery to the calf. While increased birth weights are not always reported when a protein supplement is provided during late gestation, oftentimes increased weaning weights, and increased carcass outcomes are noted. Continued efforts to understand how maternal diet impact uteroplacental blood flow, placental vascularity, and other factors associated with nutrient absorption may be key for enhancement of nutrient transfer within the reproduction tissues.
